# Measuring the pulse of trees; using the vascular system to predict tree mortality in the 21st century

**DOI:** 10.1093/conphys/coz046

**Published:** 2019-08-13

**Authors:** Timothy J Brodribb, Herve Cochard, Celia Rodriguez Dominguez

**Affiliations:** 1 School of Natural Sciences, University of Tasmania, Bag 55 ,Hobart, Tasmania, Australia; 2 PIAF,INRA,Clermont Ferrand,Auvergne,France

**Keywords:** Drought, extinction, hydraulic, tree mortality

## Abstract

Tree mortality during hot and dry conditions presents a stark reminder of the vulnerability of plant species to climatic extremes. The current global warming trend makes predicting the impacts of hot/dry events on species survival an urgent task; yet, the standard tools for this purpose lack a physiological basis. This review examines a diversity of recent evidence demonstrating how physiological attributes of plant vascular systems can explain not only why trees die during drought, but also their distributional limits according to rainfall. These important advances in the science of plant water transport physiology provide the basis for new hydraulic models that can provide credible predictions of not only how but when, where and which species will be impacted by changes in rainfall and temperature in the future. Applying a recently developed hydraulic model using realistic parameters, we show that even apparently safe mesic forest in central France is predicted to experience major forest mortality before the end of the century.

## Teaser

Tree death is a major legacy of climatic extremes, yet mechanistic models are rarely used to connect these events. In this perspective we review recent advances in tree hydraulic physiology that enable a new physiological approach to predict when, where and which species will die under future warming.

## Rising CO_2_ and photosynthesis

We know that CO_2_ levels are rising because our instruments tell us so, but plants directly sense these changes because they interact directly with CO_2_ in their everyday business. Indeed, the easiest detectable impact on plants grown under high CO_2_ is positive; an increase in photosynthesis caused by the fact that CO_2_ is the primary substrate for photosynthetic carbon fixation. Thus, plants artificially exposed to higher CO_2_ initially tend to photosynthesize faster, but over subsequent hours, months and years they reduce the entry of CO_2_ into their leaves, restoring a conservative ratio of leaf internal to external CO_2_ concentration (Franks *et al.*, 2014). This reduced entry to CO_2_ occurs because plants apparently sense CO_2_ directly, actively lowering the porosity of their leaves when CO_2_ rises, limiting the rate of CO_2_ uptake and transpiration in leaves (Engineer *et al.*, 2016). Long-term acclimation to CO_2_ has thus been linked to an increase in the efficiency of water use in photosynthesis of tree species over the past century (Ainsworth and Rogers, 2007; Peñuelas *et al.*, 2011; Frank *et al.*, 2015). Such improved conditions for efficient photosynthesis and water use at high atmospheric CO_2_ in the geological past are believed to have produced negative feedbacks that acted to stabilize atmospheric CO_2_ levels over time. Similarly there are signs that this photosynthetic buffering of CO_2_ levels is also in play under the contemporary anthropogenic CO_2_ perturbation (Ukkola *et al.*, 2016).

Improved photosynthetic conditions constitute a subtle silver lining to the current age of anthropogenic CO_2_ inundation. However, the dark cloud in this modern global CO_2_ experiment exists in the form of an associated rise in atmospheric temperature. This review will explain why a continuing temperature rise holds potentially catastrophic dangers for plant species in terms of accelerated failure of the plant vascular system during drought. We will discuss the physiology of plant failure, methods of quantifying species vulnerability and the potential to move beyond niche modelling as a means of predicting when, where and which species are in danger of mortality.

## Rising temperature as a driver of damage

The most damaging impact of rising temperature on plants is unlikely to be the direct effect of leaf warming but rather the consequences of increased transpiration on the depletion of soil water. Although increased global temperatures may increase the growing season at higher latitudes, a potentially more important global impact of greater heat is increased evaporation (Frank *et al.*, 2015). The rate of water loss from leaves is highly sensitive to temperature such that all else being equal the loss of water from a tree doubles if leaf temperature rises from 30 to 45°C. For this reason, warming atmospheric temperature substantially increases the rate of soil dehydration even if rainfall remains static. Drier soil exposes the water transport system of plants to increased water deficit, increasing the risk of catastrophic damage to the vasculature system (see below). Rapid changes in water balance therefore constitute a major problem for plants because the costs and trade-offs associated with the acquisition, conservation and transport of water have been central drivers of terrestrial plant evolution and extinction since the earliest evolution of land plants (Sperry, 2003). Thus, we find that tree species distribute according to their vascular drought resistances (Brodribb and Hill, 1999), and that the safety margins between survival or damage/mortality during drought may be rather small across the spectrum of rainfall from arid to rainforest communities (Choat *et al.*, 2012).

Increasing reports of the combined effects of extreme drought and heat producing forest mortality across the globe (McDowell *et al.*, 2008) seem to support the notion that the warming climate is likely to drive increased forest mortality into the future. To understand this pattern, it is imperative to understand the cause of mortality during drought.

**Figure 1:**
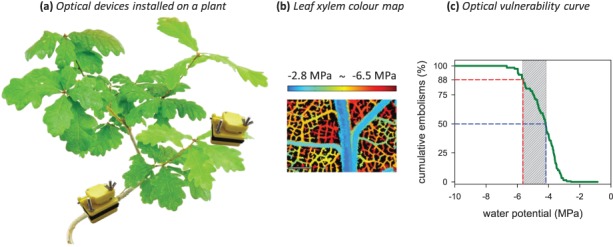
Different species have different vulnerability of xylem tissue to damage by cavitation. The ‘optical method’ (Brodribb *et al.*, 2017) is a recently developed approach for quantifying the vulnerability of plant tissues to cavitation during water stress. The optical method is applied here in a *Quercus robur* plant to visualize cavitation in stem and leaf xylem tissues. (**a**) Custom-built miniaturized cameras are shown installed on a stem and a leaf of an oak plant to monitor xylem cavitation events during plant dehydration. (**b**) Colour map depicting the location of xylem cavitation events in leaf veins and the level of water potential that induces those events (colour scale) on a *Q. robur* leaf (scale bar, 1 mm). (**c**) Optical hydraulic vulnerability curve showing the cumulative embolisms visualized against the water deficit (water potential) in the plant. Plants typically die when > 50% of xylem tissue is damaged, so P_50_ (blue dashed line) and P_88_ (red dashed line) values (i.e. water potential at which 50% and 88% of xylem vessels are embolized, respectively) describe the range of water potentials (striped grey bar) where plant mortality is likely to occur.

## Plant vascular function explains tree death during drought

The observation that water flow through plants is vulnerable to blockage under conditions of stress (Tyree and Sperry, 1988) was a transformational discovery that has enabled us the best chance yet to understand and predict the conditions that trigger tree mortality. The process of ‘hydraulic failure’ arises from the fact that plants must draw water from the soil and transport it to leaves to replace water transpired during photosynthesis. Due to the very large volumes of water required to keep leaves hydrated during photosynthesis, plants rely on hollow tubes formed by dead xylem cells that link together to connect photosynthetic tissues with a supply of water in the soil. The process of transport relies on the generation of a substantial tension in the leaves that pulls water from the soil and through the vascular system (Zimmermann, 2013). Water transport by cohesion and tension in the xylem cells works while the vascular system remains hermetically sealed against air entering through the many air/xylem interfaces that exist in a plant, but increasing tension during water stress exposes weaknesses in the membranes of the xylem, ultimately resulting in failures of the hermetic seals between the atmosphere and the xylem conduits (Crombie *et al.*, 1985). The process of hydraulic failure begins when ‘cavitation’ of the water columns within xylem cells is caused by the entry of minute bubbles dragged across the xylem membrane into the lumen where they expand under the tension (negative pressure) and permanently block the xylem conduits (Sperry and Tyree, 1988). As the xylem becomes progressively blocked, the plant is increasingly separated from water in the soil causing accelerated dehydration, increased xylem damage and a potential feedback known as ‘runaway cavitation’ whereby tissues become completely disconnected from the water supply and quickly lethally dehydrated (Tyree and Sperry, 1988). Although the final, lethal feedback in hydraulic failure loop is very hard to capture experimentally, strong associations between cavitation thresholds and tree mortality point towards this as a primary cause of tree death (see below).

Although other drought-related effects such as increased biotic damage can contribute to tree mortality during water stress (McDowell *et al.*, 2008), extensive evidence now points to cavitation and ‘hydraulic failure’ as the primary cause of drought-related mortality (Choat *et al.*, 2018). Clear links between xylem cavitation and plant mortality across a diversity of trees have been established under experimental conditions (Blackman *et al.*, 2009; Brodribb and Cochard, 2009). In addition, large-scale observations of tree damage and mortality under both natural (Anderegg *et al.*, 2016; Adams *et al.*, 2017) and artificial (Rowland *et al.*, 2015) drought indicate that hydraulic failure provides the best explanation for patterns of damage and mortality in natural forest systems.

The identification of hydraulic failure as a universal trigger of plant mortality during drought has been a great step forwards because it means that species threatened by mortality can finally be identified by their intrinsic physiology, rather than by traditional methods using climatic niche statistics that may or may not reflect their actual growth limitations. An important feature of xylem cavitation is that the ‘xylem vulnerability’ of different species to cavitation under water stress varies enormously. This variation is widely viewed as being the product of an evolutionary trade-off between the efficiency of water transport versus safety from cavitation (Gleason *et al.*, 2016). Differences in the porosity of membranes between xylem conduits are thought to explain this trade-off, with higher transport efficiency associated with more porous membranes that are less able to resist cavitation (Choat *et al.*, 2003). Despite the existence of a large range of xylem vulnerabilities among species, this trait appears to be relatively conserved within species, thus allowing the species to be characterized by their intrinsic vulnerability to xylem cavitation (Wortemann *et al.*, 2011). Thus, it is possible to measure species-specific vulnerability using excised stems or leaves to quantify the point of failure of the water transport system (Brodersen *et al.*, 2019). Techniques to quantify xylem vulnerability typically involve the dehydration of excised branches while periodically measuring the flow properties of the xylem. Although some of these techniques have recently been shown to produce errors associated with tissue excision (Longepierre *et al.*, 2014; Torres-Ruiz *et al.*, 2014), new methods have been developed that avoid excision errors by either measuring *in situ* on intact plants or by measuring leaf tissue that can be excised without significant perturbation of the vascular system. One of these methods uses x-ray computed tomography (CT) to identify the presence of air in xylem cells (Cochard *et al.*, 2015), while the other uses an optical technique (Fig. 1) to visualize the process of cavitation in xylem tissue (Brodribb *et al.*, 2016). X-ray CT imaging produces a beautifully resolved 3D map of embolism in the xylem, but it is limited by cost and temporal resolution (scans can only be made infrequently during plant dehydration). The optical method by contrast provides very high temporal resolution of cavitation in the sample but lacks the spatial precision of the CT method. However, the simplicity and low cost of the optical method make it highly suitable for relatively high-throughput measurements that could allow the characterization of forest communities (Fig. 1).

## Using xylem vulnerability to predict mortality

The potential for using variation in xylem vulnerability to predict species mortality and distribution is clearly observable from strong associations between species’ rainfall niches and their vulnerability to cavitation. In a diversity of tree species (Blackman *et al.*, 2012, 2014; Li *et al.*, 2018; Skelton *et al.*, 2018), herbaceous (Dória et al., 2018) and crop species (Johnson *et al.*, 2018; Volaire *et al.*, 2018), correlations exist whereby species growing in wet climates produce xylem that is more vulnerable to cavitation that of drier forest species. These patterns demonstrate the fundamental importance of species-specific xylem vulnerability in structuring the composition of vegetation growing at any location and climate, while indicating the likelihood of forest compositional change via mortality under conditions of changing temperature and rainfall (Anderegg *et al.*, 2018). Furthermore, these relationships between xylem vulnerability and climate underscore the great potential to use plant hydraulics to model drought-induced mortality and species distribution. The advantage of hydraulic modelling is that the basis of the model is functional, designed to capture the processes that cause damage and death to plants. A mechanistic/analytical hydraulic approach should provide advantages over empirical models that predict future distributions based on contemporary distribution, ignorant of the processes driving selection and mortality. Furthermore, the hydraulic approach is not limited by the quality of the ‘training sets’ used by niche model approaches to project contemporary plant distributions into the future. Certainly, it is more complicated to use a hydraulic model compared with a niche model because the parameterization requires knowledge of individual species physiology, but the improved confidence achieved by employing a quantitative mechanistic approach should outweigh the extra knowledge cost (in terms of gathering the physiological information required to characterize species). In terms of complexity, hydraulic models of plant mortality are relatively easy to conceive because they are structured by the behaviour of non-living xylem tissue and can thus be expected to follow relatively simple physical rules (BOX 1).

**Figure 2:**
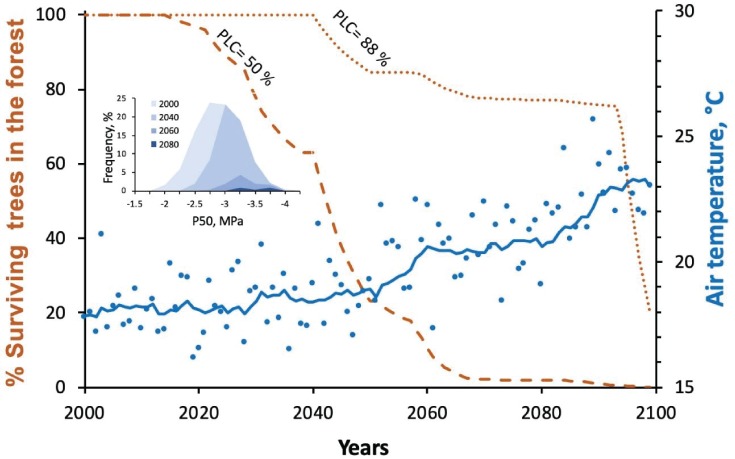
Output from a hydraulic model used to estimate the temporal erosions of population and genetic diversity of *Fagus* trees under a future climatic scenario in central France. Depending on the amount of xylem damage expected to cause mortality two scenarios for tree decline are predicted. If 50% xylem failure is lethal (dashed line) then a collapse in adult tree number is predicted after 2020 due to xylem cavitation produced by increased transpiration and soil water deficit. A more conservative rate of tree decline is predicted if plants are able to survive up to 88% loss of xylem function (dotted line). The insert shows the distribution of tree genetic sensitivity to drought (xylem vulnerability: P_50_) at four time points in the future, showing a shift to more resistant varieties. Under this RCP8.5 scenario (continuing increase in CO_2_ emissions), a dramatic collapse of tree population is predicted by the middle of this century, with most vulnerable genotypes dying first.

BOX 1 Essentially a plant can be considered as a volume of water attached the soil water by a pipeline that fails at a specified tension (species-specific). The volume of water in the soil largely determines the tension in the xylem water column, and this volume of soil water (determined by the root architecture and soil properties) is increased by rainfall and reduced by water transpired by the plant. This tractable framework can be parameterized by species-specific information about the porosity of the stomata and cuticle, root properties and of course the vulnerability of the xylem to cavitation. Thus, it is possible to run time series to determine whether plants are exposed to cavitation damage under different climatic scenarios. Due to the complexities of pinpointing the precise moment of tree death, cavitation damage is translated to tree mortality based on observations that conifers typically die after 50% stem cavitation (Brodribb and Cochard, 2009) and angiosperms 88% stem cavitation (Urli *et al.*, 2013). This 50–88% stem mortality trigger value is used as a good approximation of the water tension likely to cause widespread catastrophic embolism in branch tips because it can be easily measured, while it is much harder to precisely resolve the tension causing rapid and catastrophic failure of vascular system when 100% of conduits are cavitated. Here, we use both trigger points to examine the range of mortality expected (Fig. 2). Different xylem vulnerabilities can then be used to predict which species will be damaged or killed by droughts under specified conditions of climate and soil type. The power of this type of hydraulic mortality model has been demonstrated in manipulative pot experiments but is yet to be confirmed under natural forest conditions (Martin-StPaul *et al.*, 2017).

## Hydraulic modelling to predict future mortality

The relative simplicity of plant water transport models, coupled with the causal association between xylem damage and plant death makes this an ideal approach to gauge the magnitude of the tree mortality crisis that may be approaching as global temperatures rise. Hydraulic flow models (Sperry *et al.*, 1998) have already been used with some success to explain contemporary forest damage (Anderegg *et al.*, 2015), but there are few cases where hydraulic models have been used to predict future forest damage. To demonstrate the potential for using mechanistic models of plant hydraulics to simulate the impacts of future climatic perturbation on tree mortality we used the recently developed model SurEau (Martin-StPaul *et al.*, 2017; Cochard, 2019) to predict mortality in *Fagus sylvatica* forest in the centre of France. The model computes the daily and seasonal dynamics of tree transpiration, water potentials and xylem blockage by cavitation under different climatic time series (see BOX 1). The simulation here uses 1960–2100 daily climatic data from climatic scenario RCP8.5 generated with the GCM MPI-ESM-LR/RCM RCA4 models. The forest was assumed to be initially composed of tree genotypes displaying a variability of vulnerability to cavitation matching the observations of Wortemann *et al.* (2013) for this species. Other traits, such a leaf area, rooting depth and stomatal behaviour, were assumed to be constant across genotypes and estimated from observations, adjusted to produce no mortality (i.e. embolism < 50%) for the historical 1960–2000 time series. Tree death in the model was triggered by plants crossing a threshold of either > 50%, or a more conservative > 88% cavitation (Brodribb and Cochard, 2009). For each year and each genotype, we computed a probability of mortality based on the occurrence of lethal events during the past decade. From this we derived the temporal evolution of the number trees of each genotype in the forest. The model output shows a sharp reduction in tree population starting after 2020 and a complete forest mortality by the end of the century when mortality was set at 50% cavitation (Fig. 2). This reduction is predicted to be accompanied by an erosion of genetic diversity, the most vulnerable trees dying first (Fig. 2 insert). The more conservative hypothesis for mortality (i.e. embolism >88%) or a more optimistic climatic scenario (RCP4.5) yielded similar results but delayed in time.

## Monitoring

Hydraulic modelling holds tremendous potential as a means of predicting tree death and species extirpation at local, regional and global scales. However, maximizing this potential will demand resources to be focused towards understanding the diversity and plasticity of xylem vulnerability within and among species. At the same time, a better understanding of the relationship between rainfall and plant hydration will be achieved as arrays of sensors capable of monitoring measuring plant water potential, growth and plant water stress become distributed across forests worldwide. These efforts have already begun at local scales, identifying likely changes in forest community composition (Skelton *et al.*, 2017; Fontes *et al.*, 2018) and highlighting those species for which interventionist conservation may be a last resort. Scaling up to larger regions should be possible by combining soil and tree-based sensory arrays with airborne or spaceborne hyperspectral sensing tools. The ultimate goal of this coupled modelling-sensing approach will be the ability to monitor and ultimately predict forest heath at a global scale.

## Conclusion

The aims of this perspective have been two-fold; firstly to highlight the benefits of using the measurable attributes of the plant water transport system as a tool for understanding plant distribution and the probability of forest mortality under future climatic scenarios. The development of new techniques should enable the characterization of entire forest communities as new innovations continue to improve the efficiency of measurement. Expanded sampling of hydraulic vulnerability will enable to the identification of particularly vulnerable species and will provide a mechanistic means of understanding the limits of species distributions, enabling predictions of how geographical limits can be expected to change as global temperatures rise.

The second aim of this perspective has been to demonstrate why increasing global temperature poses a threat to tree species in terms of increasing the probability of mortality during periods of water shortage. Our simulation using hydraulic modelling indicates a high probability of extensive mortality in a mesic deciduous forest in France under a warming scenario characterized by increasing CO_2_ emissions over the century. These simulations, although preliminary, provide strong motivation to carefully evaluate the future of forests under warming climate conditions.

With knowledge of the types of data required to understand plant hydraulic stress, and process-based models to predict species exposure to future mortality we are in a good position to implement sampling and monitoring to create a picture of how forests are likely to change in the future. This knowledge will enable those engaged in forest and ecosystem management to have an informed anticipation of inevitable change.
